# PMA and Ionomycin Induce Glioblastoma Cell Death: Activation-Induced Cell-Death-Like Phenomena Occur in Glioma Cells

**DOI:** 10.1371/journal.pone.0076717

**Published:** 2013-10-09

**Authors:** Sheng Han, Xinxin Tie, Lingxuan Meng, Yunjie Wang, Anhua Wu

**Affiliations:** Department of Neurosurgery, The First Hospital of China Medical University, Shenyang, Liaoning, China; National Cancer Institute (INCA), Brazil

## Abstract

Phorbol myristate acetate (PMA) and ionomycin (Io) can induce T cell activation and proliferation. Furthermore, they stimulate activation-induced cell death (AICD) in mature lymphocytes via Fas/Fas ligand (FasL) up-regulation. In this study, we explored the influence of PMA/Io treatment on glioblastoma cells, and found that AICD-like phenomena may also occur in glioma. Using the MTT assay and cell counting, we demonstrated that treatment of PMA/Io significantly inhibited the proliferation of glioma cell lines, U87 and U251. TUNEL assays and transmission electron microscopy revealed that PMA/Io markedly induced U87 and U251 cell apoptosis. Propidium iodide staining and flow cytometry showed that treatment with PMA/Io resulted in an arrestment of cell cycle and an increase in cell death. Using real-time PCR and western blot, we found that PMA/Io up-regulated the expression of Fas and FasL at both mRNA and protein level, which confirmed that PMA/Io induced glioma cell death. Specific knockdown of NFAT1 expression by small hairpin RNA greatly reduced the PMA/Io induced cell death and apoptosis by inhibition of FasL expression. Microarray analysis showed that the expression of NFAT1 significantly correlated with the expression of Fas. The coexistence of Fas with NFAT1 *in vivo* provides the background for AICD-like phenomena to occur in glioma. These findings demonstrate that PMA/Io can induce glioblastoma cell death through the NFAT1-Fas/FasL pathway. Glioma-related AICD-like phenomena may provide a novel avenue for glioma treatment.

## Introduction

Glioblastoma multiforme (GBM) is the most aggressive type of glioma; even with combined therapy, the prognosis of GBM is still very poor [1], [2]. Using microarray analysis, we found that nuclear factor of activated T cells (NFAT)-1 is overexpressed in GBM [3]. Moreover, NFAT1 has been associated with tumor cell survival, apoptosis, migration and invasion [4], [5]. In addition, NFAT signaling can regulate cell death in many central nervous system diseases, including inflammation, tumors and degenerative diseases [6], [7], [8], [9], [10]. Therefore, we speculate that factors activating NFAT1, such as phorbol myristate acetate (PMA) and ionomycin (Io), will further influence GBM cell growth. The combination of PMA and Io has been widely used in the study of T cell activation [11], [12], [13], [14]. Through activation of protein kinase C (PKC) and calcineurin, PMA/Io can activate many transcription factors, including NF-κB and members of the NFAT family, and subsequently regulate the expression of numerous genes [13]. In resting cells, highly phosphorylated NFAT1 is in an inactive state and restricted to the cytoplasm. Activated by PMA and Io, NFAT1 is dephosphorylated, translocates to the nucleus, binds to its target promoter elements and regulates the transcription of specific responsive genes, such as Fas ligand (FasL), and cyclin A2 [15], [5]. Although PMA/Io can induce T cell proliferation, it can also promote activation-induced cell death (AICD) in lymphocytes under some specific circumstances [16], [17]. The expression of Fas and the induced-expression of FasL play a major role in this process [18], [19], [20], [21]. Recently, there are several studies that show the importance of Fas/FasL pathway in the apoptosis of glia cells and their respective tumor types [22], [23], [24], [9], [25], [26], [27]. In this study, we aimed to investigate the effect of PMA/Io administration on GBM cells and the related mechanism.

## Materials and Methods

### Cell culture

Human GBM cell lines, U87 and U251 were obtained from the Chinese Academy of Sciences cell bank (Shanghai, China). Cells were maintained in Dulbecco's modified Eagle's medium (DMEM), supplemented with 10% fetal bovine serum (FBS, Invitrogen). Cells were incubated at 37°C with 5% CO_2_.

### Antibodies and other reagents

Mouse monoclonal anti-NFAT1 antibody (Clone number 25A10.D6.D2) and rat monoclonal anti-FasL neutralizing antibody (Clone number 101624) were purchased from Abcam (Cambridge, UK). Rabbit polyclonal anti-Fas antibody (Clone number A-20) and secondary antibodies were purchased from Santa Cruz Biotechnology Inc (CA, USA). All other reagents and supplies were purchased from Sigma-Aldrich (St. Louis, MO, USA), unless otherwise stated.

### Cell proliferation assay

The 3-(4,5-dimethylthiazolyl-2)-2,5-diphenyltetrazolium bromide (MTT) assay was performed to detect cell proliferation. Briefly, cells were seeded in 96-well plates at a density of 2×10^3^ cells/well. After 24 h of incubation, cells were serum starved overnight. Cells were treated with 50 ng/mL PMA and/or 10 ng/mL Io for 24, 48, 72, 96 or 120 h. At each time point, 20 µL of 5 mg/mL MTT solution was added to each well. After 4 h of incubation, media was removed from the wells by aspiration and formazan crystals were dissolved in 150 µL of dimethyl sulfoxide (DMSO). Color intensity was measured at 490 nm with an enzyme-linked immunosorbent assay plate reader (Tecan Sunrise Remote, Austria).

### Cell counting

Cells were seeded at 5×10^3^ cells per well in DMEM with 10% FBS in 24-well plates and grown for 24 h. Then, cells were treated with 50 ng/mL PMA and/or 10 ng/mL Io for 48 h. After that, the medium was removed, cells were washed with PBS and 200 µl of 0.25% trypsin/EDTA solution was added to detach the cells, which were counted in a hemocytometer.

### TUNEL assay

Cells were treated with 50 ng/mL PMA and/or 10 ng/mL Io in 6-well plates (5×10^5^ cells/well) for 48 h. In a separate experiment, cells were pre-treated with 10 μg/mL anti-FasL neutralizing antibody for 24 h before PMA/Io administration. Then, all cells were fixed and permeabilized in 70% ethanol. The cells were stained with a commercial terminal deoxynucleotidyl transferase dUTP nick end labeling (TUNEL) kit (Merck), according to the instructions. The TUNEL-positive cells were observed under a fluorescence microscope and the cells with green fluorescence were defined as apoptotic cells. The apoptosis rate was calculated as follows: Apoptosis rate  =  Positive cells/(Positive cells+ Negative cells) ×100%.

### Transmission electron microscopic (TEM) study

After 48 h pre-treatment with 50 ng/mL PMA and 10 ng/mL Io, U87 and U251 cells were fixed with 2.5% glutaraldehyde, washed and post-fixed in osmium. Following dehydration by serial dilutions of ethanol, cells were stained with uranyl acetate, and embedded in culture dishes. Thin sections of the cured blocks were cut with a diamond knife, stained with lead citrate, and then observed and photographed under a transmission electron microscope (JEOL 1200-EX, Tokyo, Japan).

### Cell cycle and sub-G_0_ analysis

Cells treated with 50 ng/mL PMA and 10 ng/mL Io were plated in six-well microtiter plates. To assess the cell cycle and the sub-G_0_ DNA content, after 24 h or 48 h respectively, the cells were trypsinized and washed once with PBS. The cells were then stained with propidium iodide (PI, 75 μM) in the presence of NP-40. The DNA content was analyzed using flow cytometry by collecting 10,000 events for cell cycle analysis, or 15,000 events for sub-G_0_ analysis, using a FACScalibur flow cytometer and CellQuest software (BD Biosciences, San Jose, CA, USA).

### Patients and samples

Clinical glioma samples (*n* = 111) were collected from the Chinese Glioma Genome Atlas (CGGA, http://www.cgga.org.cn), including 66 primary GBMs (P), 8 anaplastic astrocytomas (AA) and 37 astrocytomas (A). All patients underwent surgical resection between January 2005 and December 2009. The histological diagnosis was established and verified by two neuropathologists according to the 2007 World Health Organization (WHO) classification guidelines. In addition, normal brain tissue samples (N = 3) from cancer-free patients who underwent surgery for primary epilepsy were also obtained from CGGA specimen bank. This study was approved by The First Hospital of China Medical University institutional review boards, and informed, written consent was obtained from all patients.

### Microarray analysis

The clinical samples were immediately snap-frozen in liquid nitrogen after resection. For each sample, the percentage of tumor cells was examined using a hematoxylin and eosin–stained frozen section before RNA extraction. Only samples with more than 80% tumor cells were qualified for analysis.

The mirVana miRNA Isolation kit (Ambion) was used for total RNA extraction, according to the manufacturer's instructions. RNA concentration and quality were assessed using a NanoDrop ND-1000 spectrophotometer (NanoDrop Technologies). A total of 111 samples (66P, 8AA, 37A) were subjected to microarray analysis using Agilent Whole Human Genome Arrays according to the manufacturer's protocol. The integrity of total RNA was checked using an Agilent 2100 Bioanalyzer. Complementary DNA and biotinylated cRNA were synthesized and hybridized to the array. An Agilent G2565BA Microarray Scanner System and Agilent Feature Extraction Software (version 9.1) were used for data acquisition. Probe intensities were normalized using GeneSpring GX 11.0 (Agilent).

### NFAT1 gene expression knockdown

NFAT1 small hairpin RNA (shRNA) plasmid and control shRNA plasmid (Santa Cruz Biotechnology) were transfected into U87 and U251 cells according to the manufacturer's protocol. Briefly, cells were seeded in a six-well plate and grown to 50–70% confluence in antibiotic-free DMEM, supplemented with 10% FBS. The cells were washed twice with 2 mL of shRNA Transfection Medium (Santa Cruz Biotechnology) before 0.8 mL of shRNA Plasmid Transfection Medium was added. After adding 200 μL shRNA Plasmid DNA/shRNA Plasmid Transfection Reagent Complex (Santa Cruz Biotechnology), the cells were incubated for 8 h at 37°C with 5% CO_2_. Subsequently, 1 mL of DMEM with 20% FBS was added. At 48 h post-transfection, the medium was replaced with fresh medium containing 5 μg/mL puromycin for selection of stably transfected cells. The medium was changed every 2 days. Four days later, NFAT1 gene expression was visualized using RT-PCR and/or western blot.

### RT-PCR

Total RNA was isolated from U87, U87-control-shRNA and U87-NFAT1-shRNA cells using TRIzol reagent (Invitrogen), in accordance with the manufacturer's protocol. Total RNA was reversely transcribed into cDNA and used for PCR amplification. Specific primers for NFAT1 and GAPDH were: NFAT1 forward: 5′-CGG GCC CAC TAT GAG ACA GAA-3′ and NFAT1 reverse: 5′-GCT CAT CAG CTG TCC CAA TGA A-3′; GAPDH forward: 5′-GCA CCG TCA AGG CTG AGA AC-3′ and GAPDH reverse: 5′-TGG TGA AGA CGC CAG TGG A-3′ (TaKaRa), respectively. The reactions were carried out with a polymerase-activating step of 95°C for 10 min, followed by 40 cycles of a two-step cycling program (95°C for 15 s; 60°C for 1 min). PCR products were electrophoresed on a 1% agarose gel containing ethidium bromide.

### Immunofluorescence

Cells grown on coverslips were treated with 50 ng/mL PMA or 10 ng/mL Io for 60 min. Subsequently, cells were washed, fixed, blocked and probed with the anti-NFAT1 antibody (1∶100). NFAT1 was detected with a fluorochrome-conjugated secondary antibody, and nuclei were counterstained with Hoechst 33342. Coverslips were mounted on glass slides, and cells were visualized with a confocal microscope (Olympus FV1000S-SIM, Japan). An image analyses software, Image pro plus 6.0 (Media Cybernetics CO., American), was used for digital photographs analysis to semi-quantify the mean intensity of NFAT1 fluorescence in the nucleus.

### Western blot analysis

To investigate the protein level of NFAT1, a total cell protein extraction kit (Milipore, Billerica, MA, USA) was used to extract total protein from normal brain tissues, glioma tissues, U87 and U251 cells. In another experiment, cells were treated with 50 ng/mL PMA and 10 ng/mL Io for 24 h, and then total cell protein was extracted to examine the protein level of Fas/FasL. Protein concentrations were determined using the Coomassie protein assay (Bradford). An equivalent amount of protein from each sample was electrophoresed by 12% SDS-PAGE and transferred to nitrocellulose membrane. After being blocked, membranes were incubated with primary antibody (1∶1000) overnight at 4°C. Membranes were then washed three times with PBS/0.1% Tween-20 (5 min each), and incubated with a corresponding secondary anybody (1∶5000) for 2 h at room temperature. Bands were detected using a chemiluminescence ECL kit (Santa Cruz Biotechnology), and were quantified using the Sigma-Gel software (Jandel Scientific Software, Sari Kafael, CA, USA).

### Real-time RT-PCR

To quantify Fas/FasL mRNA expression, real-time PCR was performed. Sequences for the primers were as follows: for Fas forward CAA GGG ATT GGA ATT GAG GA and reverse TGG AAG AAA AAT GGG CTT TG; for FasL forward TGG GGA TGT TTC AGC TCT TC and reverse CAG AGG CAT GGA CCT TGA GT. A total of 200–500 ng mRNA of each sample was used to synthesize cDNA. Amplification of cDNA was done in triplicate. PCR conditions were as follows: 1 cycle of 95°C for 3 min, followed by 45 cycles of 95°C for 20 s, 63°C for 20 s, and 72°C for 20 s.

### Cluster analysis and statistical analysis

The mRNA expression of NFAT1 and Fas obtained from microarray analysis (the microarray dataset can be accessed at this website http://www.cgga.org.cn/medical.php?mod=search, and the username is “mrnauser” and the password is “mrnausername”) were used for cluster analysis (Cluster 2.20) and Pearson correlation analysis (SPSS 13.0). Cluster analysis was performed using the hierarchical clustering method with average linkage. The results of cluster analysis were visualized using TreeView software. Chi-square test and Student's *t*-test were used to determine significance. All data are presented as the mean ± standard error of at least three independent experiments. A two-tailed *P*-value of <0.05 was regarded as statistically significant.

## Results

### PMA and Io inhibit GBM cell proliferation

**Figure 1:**
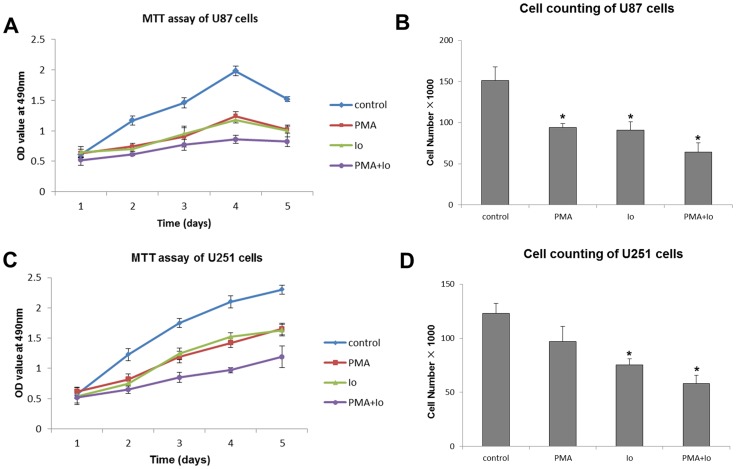
Phorbol myristate acetate (PMA) and ionomycin (Io) inhibit glioblastoma multiforme (GBM) cell proliferation. Proliferation was assessed by MTT assay and cell counting. The cells were plated in triplicate, treated with 50/mL PMA and/or 10 ng/mL Io, and analyzed for proliferation for 120 h in MTT assay and for 48 h in cell counting. The graphs are representatives of three independent experiments. (A, B) PMA and/or Io treatment significantly inhibited U87 cell proliferation. (C, D) PMA and/or Io also markedly suppressed the proliferation of U251 cells. *P<0.05 (compared with the control).

Using the MTT assay, which reflects total number of live cells, we examined the effect of PMA and/or Io treatment on U87 and U251 cell proliferation at indicated time points. As shown in **Figure 1A** and **C**, both PMA and Io significantly inhibited the proliferation of U87 and U251 cells, especially at day 3 and day 4. Moreover, the combination of PMA and Io showed the strongest inhibitory effect (*P*<0.05).

To confirm the results of MTT assay, we counted the cells after 48 h of treatment with PMA and/or Io in a hemocytometer. The presence of PMA and Io caused a significant reduction in the number of cells (**Figure 1B ** and ** D**).

### PMA and Io induce GBM cell apoptosis

**Figure 2:**
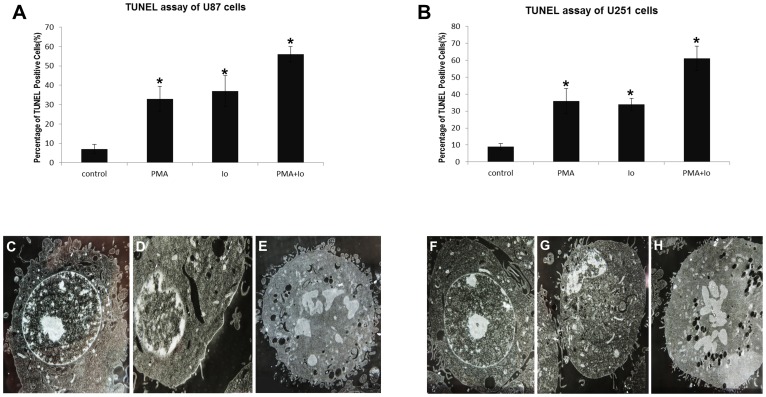
PMA and Io induce GBM cell apoptosis. Apoptotic cells were examined 48/or Io treatment in U87 and U251 cells. (A, B) Quantitative analysis of the apoptotic cells after treatment with PMA and/or Io; apoptotic cells were determined by the TUNEL assay. The graphs are representative of three independent experiments. (C–H) Representative photomicrograph (5000×) of U87 cells (C–E) and U251 cells (F–H) using transmission electron microscopy. In the control groups (C, F), the nuclear shape is normal and the nucleolus is clear. In contrast, nuclear shape changes, and condensation of chromatin and nuclear fragmentation can be observed in treated cells (D, E, G, H). *P<0.05 (compared with the control).

In order to ascertain the induction of GBM cell death by PMA and Io, the TUNEL assay, which shows characteristic chromatin condensation and nuclear fragmentation resulting from apoptotic signaling cascades, was performed. The results showed that the rate of apoptosis increased in response to PMA or Io administration, both in U87 and U251 cells (*P<*0.05). PMA combined with Io exhibited the greatest apoptotic effect (**Figure 2A, B**).

Next, nuclear changes during apoptosis induced by PMA and Io in U87 and U251 cells were examined using TEM. TEM studies showed that the nuclear shape changed in treated cells compared to control cells, while the nuclear volume decreased markedly. Characteristic alterations, including condensation of chromatin and nuclear fragmentation, can also be observed in treated cells (**Figure 2C–H**). Together, these results indicated that PMA and Io induced apoptosis of GBM cells.

### PMA and Io induce cell cycle arrest and cell death in GBM cells

**Figure 3:**
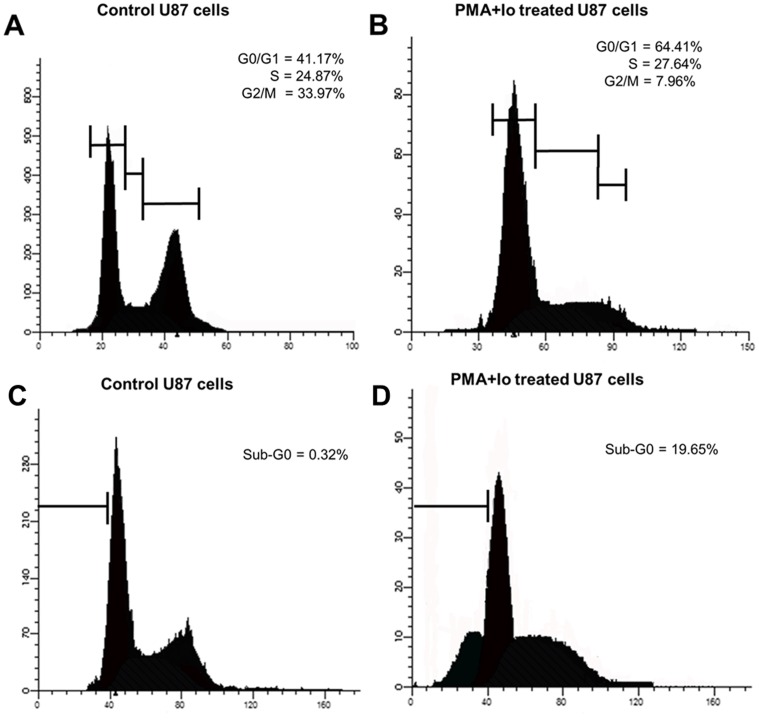
PMA and Io induce cell cycle arrest and apoptosis in U87 GBM cells. U87 cells were stained with propidium iodide (PI) and analyzed by flow cytometry for cell cycle and death. The graphs are representative of three independent experiments. (A, B) Cell cycle was analyzed by the incorporation of PI. The cells were plated in triplicate and analyzed 24 h after treatment. The percentage of cells in each phase of the cell cycle is indicated in the graphs. PMA and Io significantly arrested cells at G_0_/G_1_ phase. (C, D) Analysis of cell death 48 h after treatment. The percentage of cells in sub-G_0_ is shown in the graph.

Subsequently, to examine whether the low proliferation levels observed in PMA and Io treated cells reflects a reduction in the number of cells entering the cell cycle, we assessed the cell cycle profile 24 h after treatment by PI staining. Cell cycle analysis revealed that PMA and Io treatment significantly arrested cells at the G_0_/G_1_ phase of the cell cycle (**Figure 3A–B**).

In order to evaluate DNA fragmentation, the sub-G_0_ DNA content of U87 cells treated with PMA and Io was examined by PI staining. As shown in **Figure 3C–D**, control cells showed a low percentage of cells with sub-G_0_ DNA content. In addition, 48 h after treatment, approximately 20% of the PMA and Io-treated cells had sub-G_0_ DNA content, suggesting that PMA and Io treatment induce U87 cell death. Together, these data indicate that PMA and Io induce cell cycle arrest and cell death in GBM cells.

### The expression of NFAT1 correlated with the expression of Fas

**Figure 4:**
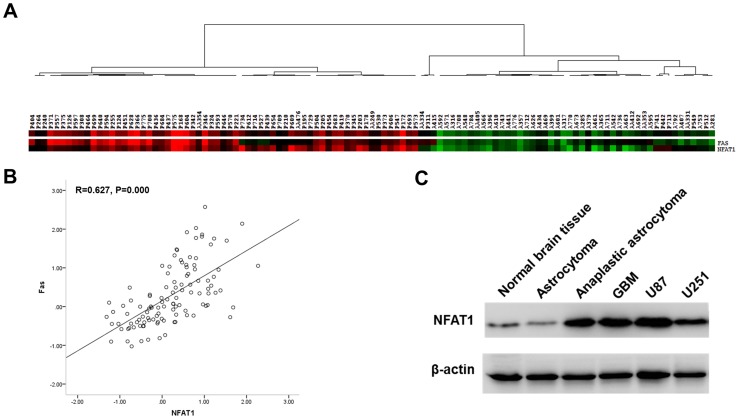
The mRNA expression of NFAT1 and Fas obtained from microarray analysis in 111 clinical samples was analyzed by cluster analysis and Pearson correlation analysis. (A) NFAT1 was overexpressed in high-grade gliomas. (A, B) The expression of NFAT1 significantly correlated with the expression of Fas in gliomas. (C) Western bot showed that NFAT1 was highly expressed in GBM clinical samples as well as in U87 and U251 cells, compared with low-grade glioma and normal brain tissue samples. The graph is a representative of three independent experiments.

**Figure 5:**
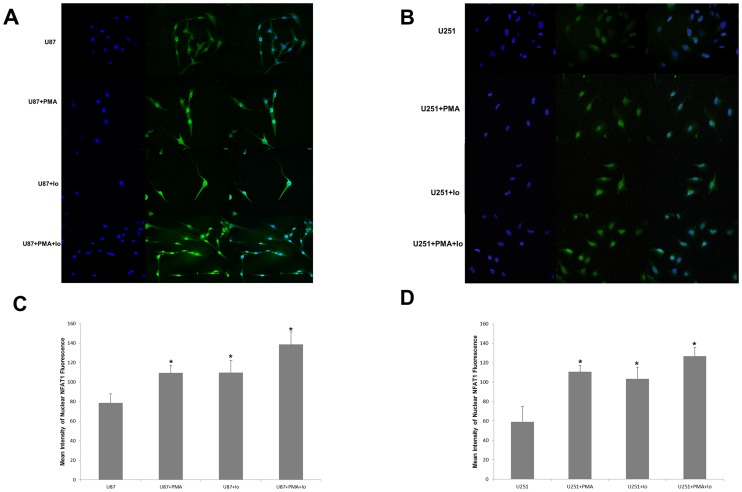
NFAT1 can be activated by PMA and Io in GBM cells. The graphs are representative of three independent experiments. (A, B) Immunofluorescent staining demonstrated that in U87 and U251 cells, NFAT1 is hyper-activated by PMA and Io treatment. Treatment of U87 and U251 cells with PMA and/or Io for 60 min caused rapid activation and nuclear translocation of NFAT1. (C, D) Semi-quantification of the mean intensity of NFAT1 fluorescence in the nucleus of U87 (C) and U251 (D) cells using Image pro plus software. *P<0.05 (compared with the control U87 and U251 cells respectively).

Using microarray analysis, we found that NFAT1 is overexpressed in GBM, compared with low-grade gliomas (**Figure 4A**). Confirming the results from the microarray analysis, western blot analysis showed a high protein level of NFAT1 in GBM U87, and U251 cells, as well as in GBM clinical samples, compared with low-grade glioma and normal brain tissue samples (**Figure 4C**). Moreover, immunofluorescent staining detected that NFAT1 can be hyper-activated by PMA and Io treatment in GBM cells. As shown in **Figure 5**, treatment of U87 and U251 cells with PMA and/or Io for 60 min caused rapid activation and nuclear translocation of NFAT1.

The mRNA expression of NFAT1and Fas obtained from microarray analysis in 111 clinical samples was analyzed with cluster analysis and Pearson correlation analysis. The expression of NFAT1 was significantly correlated with that of Fas (*R* = 0.627, *P*<0.01) in gliomas (**Figure 4B**).

### NFAT1 down-regulation prevents PMA and Io-induced cell death

**Figure 6:**
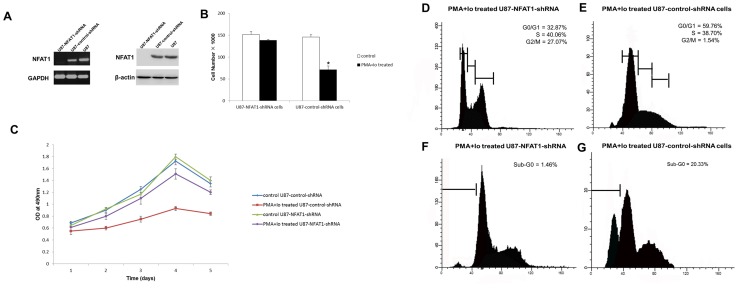
NFAT1 down-regulation prevents PMA and Io-induced cell cycle arrest and cell death. The graphs are representative of three independent experiments. (A) The expression of NFAT1 was effectively knocked down in U87 cells by transfection of specific small hairpin RNA. Cell counting (B) and MTT assay (C) showed that the knockdown of NFAT1 prevented the inhibition of proliferation by PMA and Io. (D–G) Cells were stained with PI and analyzed by flow cytometry for cell cycle and death. (D, E) Cell cycle was analyzed by the incorporation of PI. The cells were plated in triplicate and analyzed 24 h after treatment. The percentage of cells in each phase of the cell cycle is indicated in the graphs. Down-regulation of NFAT1 markedly prevented cell cycle arrest induced by PMA and Io. (F, G) Analysis of cell death 48 h after treatment. The percentage of cells in sub-G_0_ is shown in the graph. Silence of NFAT1 also prevented PMA and Io-induced U87 cell death. *P<0.05 (compared with the U87-control-shRNA cells).

**Figure 7:**
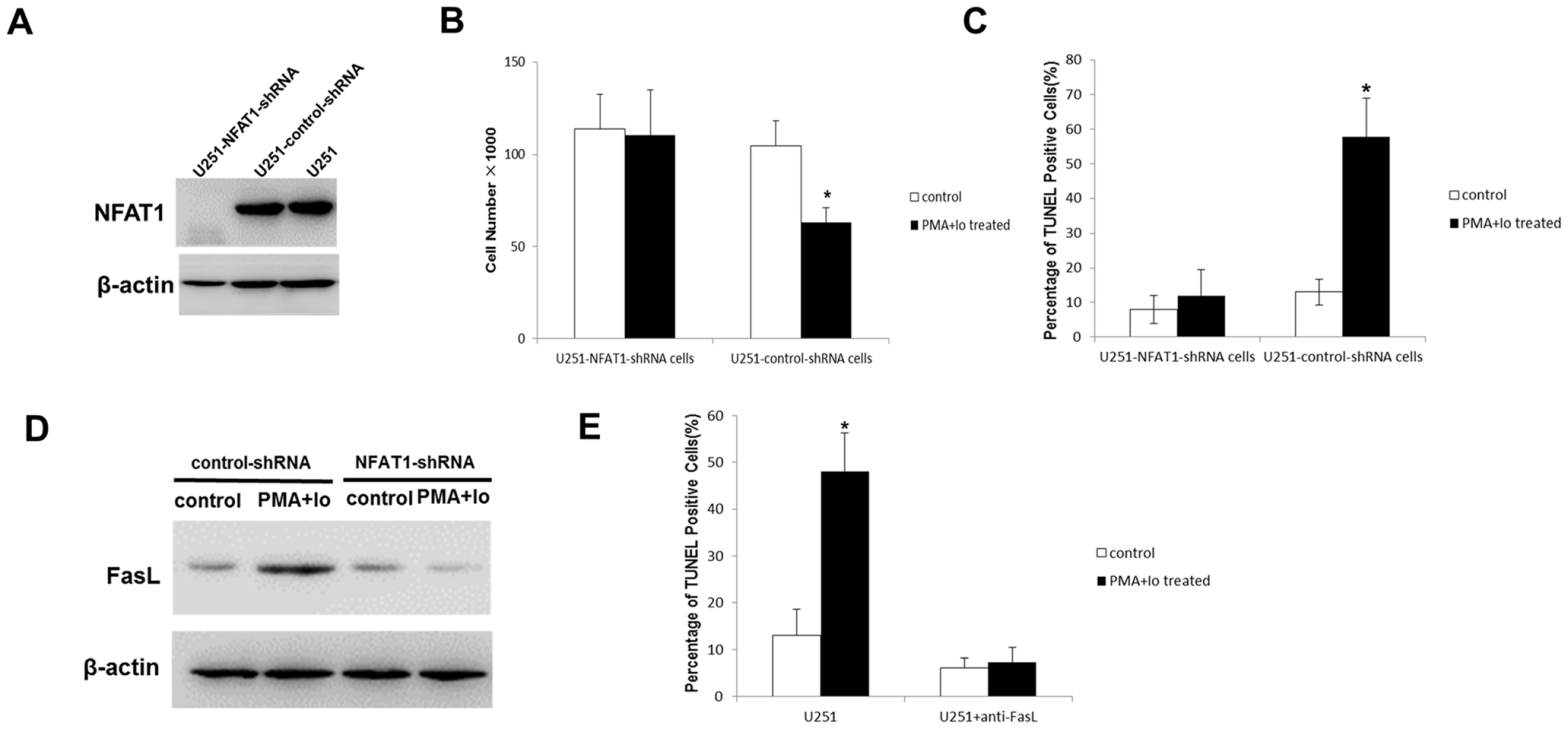
PMA and Io-induced apoptosis is NFAT1-dependent in U251 cells. The graphs are representative of three independent experiments. (A) The expression of NFAT1 was effectively knocked down in U251 cells by transfection of specific small hairpin RNA. (B) The knockdown of NFAT1 prevented the inhibition of proliferation by PMA and Io in U251 cells. (C) The TUNEL assay showed that NFAT1-silencing inhibited the apoptosis induced by PMA and Io. (D) The induction of FasL by PMA/Io was NFAT1-dependent. (E) The TUNEL assay showed that FasL neutralization by a specific anti-FasL antibody significantly inhibited PMA and Io-induced apoptosis in U251 cells. *P<0.05 (compared with the control respectively).

We used shRNA technology to silence NFAT1 gene expression to test whether it was implicated in PMA and Io-induced GBM cell death. For that purpose, NFAT1-shRNA and a transfection control (control-shRNA) were introduced into U87 and U251 cells. As shown in **Figure 6A** and **Figure 7A**, the expression of NFAT1 was effectively knocked-down in U87-NFAT1-shRNA cells and U251-NFAT1-shRNA cells.

**Figure 8:**
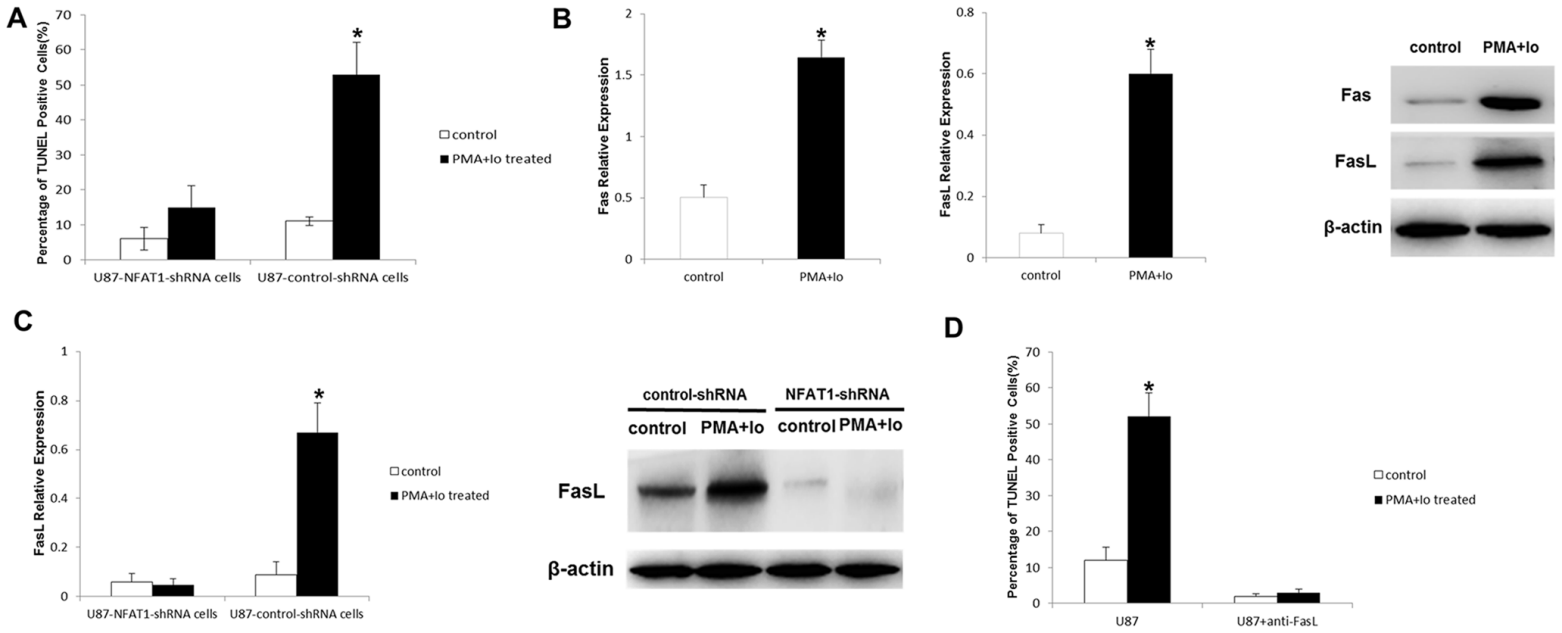
PMA and Io-induced apoptosis is NFAT1-dependent in U87 cells. The graphs are representative of three independent experiments. (A) The TUNEL assay showed that NFAT1-silencing inhibited the apoptosis induced by PMA and Io. (B) Real-time PCR and western blot analysis demonstrated that treatment of PMA and Io elevated the expression of Fas and FasL. (C) However, the induction of FasL was NFAT1-dependent. (D) The TUNEL assay showed that FasL neutralization by a specific anti-FasL antibody significantly inhibited PMA and Io-induced apoptosis. *P<0.05 (compared with the control respectively).

U87-control-shRNA cells stimulated with PMA and Io led to a reproducible decrease in proliferation. However, in the presence of NFAT1-shRNA, proliferation almost returned to control levels (**Figure 6B–C**). We then proceeded to test NFAT1 implication in PMA and Io-induced cell cycle arrest and cell death in U87 cells by flow cytometry. Following PI staining, U87-control-shRNA cells treated with PMA and Io showed a significant cell cycle arrest at G_0_/G_1_ phase after 24 h, and a marked increase of cells with sub-G_0_ DNA content after 48 h. In contrast, NFAT1-silencing greatly reduced this effect of PMA and Io in U87 cells (**Figure 6D–G**). Furthermore, the TUNEL assay revealed that although treatment of PMA and Io remarkably elevated apoptosis rate in U87-control-shRNA cells, NFAT1-silencing lowered the apoptosis rate to near control levels (**Figure 8A**). Similar results were obtained in U251 cells (**Figure 7B–C**). These findings imply a direct role of NFAT1 in PMA and Io-induced cell death in GBM cells.

### PMA and Io increase the expression of FasL in a NFAT1-dependent manner

We next set out to characterize the target genes induced by PMA and Io that contribute to promoting apoptosis in GBM cells. As shown in **Figure 8B**, 24 h after PMA and Io administration, the expression of FasL increased by 5-fold in U87 cells at both the mRNA and the protein level, which may be responsible for the apoptosis induced by PMA and Io treatment. The expression of Fas in U87 cells was also increased after the treatment of PMA and Io.

It was reported that there is a NFAT1 binding site in the promoter of the FasL gene [28]; in this research, we confirmed that, following NFAT1-silencing, the expression of FasL decreased in U87 cells. PMA and Io stimulation significantly elevated the expression of FasL in U87-control-shRNA cells, but not in U87-NFAT1-shRNA cells. FasL decreased 5.7-fold in U87-NFAT1-shRNA cells compared to U87-control-shRNA cells after stimulation with PMA and Io (**Figure 8C**). These results were confirmed in U251 cells (**Figure 7D**). These findings suggest that elevation of FasL by PMA and Io is mainly NFAT1 dependent.

### FasL neutralization inhibits PMA and Io-induced apoptosis

To further determine the role of FasL in PMA and Io-induced apoptosis, U87 and U251 cells were pre-treated with anti-FasL neutralizing antibody before PMA and Io administration. In the subsequent TUNEL assay, we found that FasL neutralization significantly inhibited PMA and Io-induced apoptosis (**Figure 7E** and **Figure 8D**). Thus, in GBM cells, PMA/Io induces apoptosis mainly through the Fas/FasL pathway, similar to AICD in lymphocytes.

## Discussion

GBM is highly malignant and resistant to currently available therapies. Despite surgery, radiotherapy and alkylating agent-based chemotherapy, the median survival time is only about 14 months [29], [30], [31]. One of the dilemmas of current combined treatment strategies is their neglect of tumor-related or treatment-related immunosuppression [30], [32], [33]. Given that the systemic reaction induced by the tumor already compromises the immune system [34], [35], [36], the immunity of GBM patients who receive radiotherapy and chemotherapy could be further damaged by these treatments. Immunosuppression not only causes lethal complications, such as severe infection, but also prompts tumor progression and impairs anti-tumor immunotherapy [37]. Thus, an agent that can both inhibit tumor growth and protect the immune system is of great therapeutic interest.

PMA and Io are routinely used to establish a T cell receptor-independent model to study T cell activation and proliferation [14]. By mimicking the phospholipase C-driven activation of PKC and the increase of cytosolic Ca^2+^, PMA and Io activate the transcription factors NFAT1, NF-κB and activator protein-1 (AP-1), and subsequently regulate downstream gene expression [38]. Under different circumstances, these agents can either activate T cells or initiate AICD in lymphocytes [17], as determined by the activated transcription factors and transcribed genes. NFAT1 plays a key role in this process [39].

We previously demonstrated that NFAT1 is overexpressed in GBM and related to the expression of IL13RA2 [40]. Moreover, we showed that NFAT1 is associated with the invasion of GBM cells [3]. Consequently, prior to the present study, we hypothesized that PMA/Io may hyper-activate NFAT1 and promote GBM growth. There is evidence that PMA and Io may be carcinogens that promote tumor cell growth by activation of PKC pathway [41], [42]. However, in the present study, we found that PMA and Io significantly inhibit proliferation and induce apoptosis in GBM cells, as evidenced by cell features such as cell cycle arrest and DNA fragmentation, which is similar to the AICD phenomena in lymphocytes.

Since PMA/Io can activate numerous signaling pathways, we hypothesized that this apoptotic effect may not be through the NFAT1 pathway. However, in contrast, when we specifically knocked down NFAT1 expression, the GBM cell death induced by PMA/Io was significantly suppressed. Therefore, NFAT1 plays a direct role in PMA/Io-induced GBM cell death. The results of this study, and our previous studies [3], suggest that the activation status of NFAT1 determines its effect in GBM cells. Partially activated NFAT1 regulates GBM cell invasion, but not proliferation. However, after being stimulated by PMA/Io, NFAT1 is hyper-activated (as shown by immunofluorescent staining) and subsequently induces cell death. This phenomenon is similar to AICD in lymphocytes. During lymphocyte activation, NFAT1 is also activated, which leads to lymphocyte proliferation [15]. Apoptosis occurs when PMA/Io further stimulates lymphocytes [16]. Our results showed that AICD-like phenomena maybe also exist in GBM cells. Therefore, we next investigated whether PMA/Io-induced GBM cell death shares a similar mechanism with that of AICD.

It has been reported that PMA/Io-induced AICD is mainly through up-regulation of FasL expression [43]. In this study, we found that PMA/Io induced high expression of FasL, both at the mRNA and the protein level. When a specific antibody neutralized FasL, PMA/Io-induced GBM cell death was significantly inhibited. We also demonstrated that the induction of FasL by PMA/Io is substantially NFAT1-dependent. Thus, the mechanism of PMA/Io-induced GBM cell death is similar to that of AICD in lymphocytes. Our microarray analysis demonstrates that NFAT1 and Fas are simultaneously highly expressed in the majority of GBM. In these tumors, PMA/Io stimulation may elevate FasL expression and lead to AICD-like cell death. Moreover, previous studies identify that Fas/FasL pathway plays an important role in the apoptosis of glia cells and gliomas [25], [26], [27]. As such, induction of AICD-like cell death may be a feasible mode for the treatment of GBM.

AICD-like cell death induced by NFAT1 hyper-activation may exist in other tumors. For example, over-activation of NFAT1 has also been demonstrated to induce cell death in neuroblastomas and Burkitt's lymphomas [44], [45], [9]. However, our results suggest that overexpression of NFAT1 is necessary, but not sufficient to induce cell death on its own. Factors such as PMA/Io, which can hyper-activate NFAT1 pathways, are also required. Subsequent induction or up-regulation of Fas/FasL expression is essential. Nevertheless, overexpression of NFAT1 has been found in several cancer cells, including breast cancer and pancreatic ductal carcinoma [46], [47]. Therefore, it is possible that AICD-like cell death could be induced in these tumors. Further investigation is necessary to understand whether AICD-like cell death is a common phenomenon in tumors, as well as whether induction of AICD-like cell death can be used for the treatment of tumors.

## Conclusions

Our results demonstrate that NFAT1 hyper-activation by PMA and Io significantly induces GBM cell death through the Fas/FasL pathway, which is therapeutically significant. Agents like PMA and Io, which can effectively activate T cells and induce AICD-like tumor cell death, should be further investigated for the treatment of malignant gliomas.
